# Changes in functional properties and 5-HT modulation above and below a spinal transection in lamprey

**DOI:** 10.3389/fncir.2014.00148

**Published:** 2015-01-20

**Authors:** Matthew I. Becker, David Parker

**Affiliations:** Department of Physiology, Development and Neuroscience, University of CambridgeCambridge, UK

**Keywords:** spinal cord, neuromodulation, spinal cord injury, lamprey, 5-HT

## Abstract

In addition to the disruption of neural function below spinal cord injuries (SCI), there also can be changes in neuronal properties above and below the lesion site. The relevance of these changes is generally unclear, but they must be understood if we are to provide rational interventions. Pharmacological approaches to improving locomotor function have been studied extensively, but it is still unclear what constitutes an optimal approach. Here, we have used the lamprey to compare the modulatory effects of 5-HT and lesion-induced changes in cellular and synaptic properties in unlesioned and lesioned animals. While analyses typically focus on the sub-lesion spinal cord, we have also examined effects above the lesion to see if there are changes here that could potentially contribute to the functional recovery. Cellular and synaptic properties differed in unlesioned and lesioned spinal cords and above and below the lesion site. The cellular and synaptic modulatory effects of 5-HT also differed in lesioned and unlesioned animals, again in region-specific ways above and below the lesion site. A role for 5-HT in promoting recovery was suggested by the potential for improvement in locomotor activity when 5-HT was applied to poorly recovered animals, and by the consistent failure of animals to recover when they were incubated in PCPA to deplete 5-HT. However, PCPA did not affect swimming in animals that had already recovered, suggesting a difference in 5-HT effects after lesioning. These results show changes in 5-HT modulation and cellular and synaptic properties after recovery from a spinal cord transection. Importantly, effects are not confined to the sub-lesion spinal cord but also occur above the lesion site. This suggests that the changes may not simply reflect compensatory responses to the loss of descending inputs, but reflect the need for co-ordinated changes above and below the lesion site. The changes in modulatory effects should be considered in pharmacological approaches to functional recovery, as assumptions based on effects in the unlesioned spinal cord may not be justified.

## Introduction

Spinal cord injury is associated with a loss of sensory and motor function below a lesion site due to damage to ascending and descending tracts and local circuitry, as well as disturbances of autonomic function. There is currently no effective intervention to overcome the effects of SPI (Verma et al., 2008). A major focus is on promoting the regeneration of axons across lesion sites. This occurs spontaneously in lower vertebrates (Tanaka and Ferretti, 2009), where it is assumed to account for the functional recovery that can occur in these systems. However, in addition to loss of descending and ascending inputs across the lesion site, there are also various functional changes below the lesion site in animal models and in the human spinal cord (Cohen et al., 1988; McClellan, 1994; Bennett et al., 2001; Edgerton et al., 2001; Pearson, 2001; Rossignol et al., 2001; Wolpaw and Tennissen, 2001; Li and Bennett, 2003; Grasso et al., 2004; Li et al., 2004; Harkema, 2008; Cooke and Parker, 2009; Boulenguez et al., 2010; Rossignol and Frigon, 2011; Roy et al., 2012; Vajn et al., 2014). The role of these changes is generally unclear: they could facilitate regeneration strategies by compensating for the reduction of descending inputs (e.g., by changing functional properties to allow a reduced number of descending inputs to evoke the same functional effect as in the unlesioned cord); or they could complicate these approaches by requiring regenerated inputs to interact appropriately with varying sub-lesion networks (Nahar et al., 2012).

Pharmacological approaches to restoring function after spinal injury have been attempted in experimental and clinical settings (Rossignol et al., 2001; Tillakaratne et al., 2002; Barbeau and Norman, 2003; Parker, 2005). There is a vast literature on drug effects but still little insight into what constitutes an optimal pharmacological approach. This is complicated by the diverse neuron and state-specific effects of modulators, novel effects caused by interactions between modulatory systems (see Katz and Edwards, 1999), and the general difficulty of linking cellular effects to network outputs and behavior. Among the various transmitter systems, 5-HT has arguably been studied most extensively. 5-HT has significant effects on locomotion and sensory processing in the unlesioned spinal cord (Schmidt and Jordan, 2000), and there is evidence that constitutive activation of 5-HT_2_ receptors (Murray et al., 2010; D’Amico et al., 2013) or the application of 5-HT receptor agonists (see Gimenez y Ribotta et al., 1998; Hains et al., 2001; Hochman et al., 2001; Antri et al., 2003; see Gackière and Vinay (2014) for a recent review) can improve locomotor function after injury. However, the mechanisms underlying any improvements, which would ideally be targeted to improve functional recovery, are unclear.

The larval and juvenile adult lamprey has been used as a model system for studying the recovery of locomotor function after spinal cord lesions. These studies have largely focused on regeneration, which occurs to a comparable extent in both developmental stages (Cohen et al., 1988; Lurie and Selzer, 1991; McClellan, 1994; Oliphint et al., 2010), but changes in the anatomy and functional properties of spinal cord neurons have also been examined (see Yin et al., 1987). Our previous analyses have shown that cellular and synaptic properties of larval motor neurons and spinal interneurons are altered below the lesion site (Cooke and Parker, 2009). Proprioceptive feedback is also potentiated after lesioning, and the modulation of proprioceptive inputs by GABA and somatostatin is altered (Hoffman and Parker, 2011; Svensson et al., 2013).

The lamprey offers a simpler system in which to examine injury-induced changes, and ultimately try and establish their influence on recovery. Here we have examined potential lesion-induced differences in modulation by comparing the effects of 5-HT in unlesioned animals and in lesioned animals. We have extended our analyses to examine effects in young adults. While recovery in adult lampreys has been studied (Cohen et al., 1989), most studies have focused on larval animals. Given that insight is needed into how the mature nervous system responds to injury, we felt that extending our analysis to, albeit juvenile, adult animals was a useful addition. We have also examined effects below and above the lesion site. Changes below the lesion site are routinely examined, but above lesion effects have received relatively little attention (see Grasso et al., 2004). While changes below the lesion site could be an attempt to compensate for the loss of descending inputs, changes above the lesion site may also result from the loss of ascending inputs, or the need to adjust supra-lesion activity to the changes that occur below the lesion site. While in many cases preliminary, the results suggest differences in functional properties and their modulation by 5-HT after lesioning that occur in region-specific ways above and below the lesion site.

## Materials and methods

Juvenile adult lampreys (*Pertomyzon marinus*) between 100–130 mm were obtained from commercial suppliers (Acme Lamprey Company, Maine, USA). All experiments were performed under license and conformed to the requirements of the UK Home Office Animals (Scientific Procedures) Act 1986. For spinal cord transections animals were anesthetized by immersion in MS-222 (300 mg/ml, pH adjusted to 7.4) and a ~5 mm dorsal incision was made approximately 1 cm below the last gill to expose the spinal cord. The spinal cord was transected with iridectomy scissors and the incision site sealed with tissue glue (Vetbond). Following transection animals were kept at 20°C for 8–10 weeks (unlesioned animals were also kept at this temperature): at this time most animals had recovered locomotor function (McClellan, 1994) and the incision site had healed completely.

Video and electromyogram (EMG) recordings were made from unlesioned animals and animals 8–10 weeks after lesioning. EMG recordings were made by inserting bipolar Teflon insulated electrodes (0.075 mm diameter) into the muscle under MS-222 anesthesia. The electrodes were inserted approximately 0.5 cm rostral and caudal to the lesion site. After recovery from anesthesia muscle activity was recorded as the animals swam in a plastic aquarium (29 × 23.5 × 5.5 cm). Swimming activity was initiated by lightly pinching the tail or head of the animal using serrated forceps: stimulation was given 30 s after the end of the previous swimming episode. A qualitative assessment of the functional recovery was made from video recordings based on the scale derived by (Ayers (1989); see Cooke and Parker, 2009; Hoffman and Parker, 2011). EMG and extracellular activity was recorded using an A-M Systems 1700 Differential AC amplifier. All data acquisition and analysis was done using a computer equipped with an analog to digital interface (Digidata 1322A, Molecular Devices) and pClamp9 software (Molecular Devices). From the EMG of each animal a quantitative analysis of swimming was performed by measuring the episode length (duration of EMG activity), the cycle period, the coefficient of variation of bursting, and the intersegmental phase lag. Lampreys undergo a change in body size during development. However, the spinal cord represents ~80% of the total body length in each of the life cycle stages (Ruiz et al., 2004). A linear function can fit the relationship of segment to body length, where each of the assumed 100 spinal segments along the spinal cord increases proportionally with body length (Ruiz et al., 2004):

y=0.0066x+0.058

*y* is the segment length and x the total body length (Ruiz et al., 2004). Animals were measured from the tip of the oral hood to the end of the tail. To determine intersegmental phase lag (Φ), the following equation was used (Boyd and McClellan, 2002):

ϕ=[d/T]/N

*d* is the interval between rostral and caudal bursts in the same cycle on the same side; *T* is the cycle time, which is inversely proportional to the bursting frequency; *N* is the number of intervening segments which is divided by *y*.

The modulation of swimming was assessed by applying 5-HT (10–500 μM) to the aquarium water: plateau effects on the episode length and coefficient of variation (CV; standard deviation of the cycle duration divided by the mean cycle duration), aspects where significant effects of 5-HT were seen, occurred at a concentration of 200–400 μM (see Figures 8A,B; results presented in Figure 9 show only the effects of 500 μM 5-HT). While we do not know the final concentration of 5-HT in the spinal cord, we are confident that this application route allows access to the CNS as there were changes in the swimming behavior of lesioned and unlesioned animals. To examine the role of 5-HT in locomotor function and recovery we incubated animals in p-Chlorophenylalanine (PCPA; 0.006 g in 200 ml of water equivalent to 150 μM), which depletes 5-HT (Hashimoto and Fukuda, 1991; Airhart et al., 2012). Unlesioned animals were also examined after 72 h in PCPA: in this case there were marked effects on behavior that again suggest access to the CNS through this administration route. Lesioned animals were incubated for 6 weeks (PCPA was changed every 3 days), and then placed in normal aquarium water for at least two weeks to avoid acute effects of PCPA (in unlesioned animals swimming behavior had recovered within 3–5 days after removal from PCPA).

**Figure 1:**
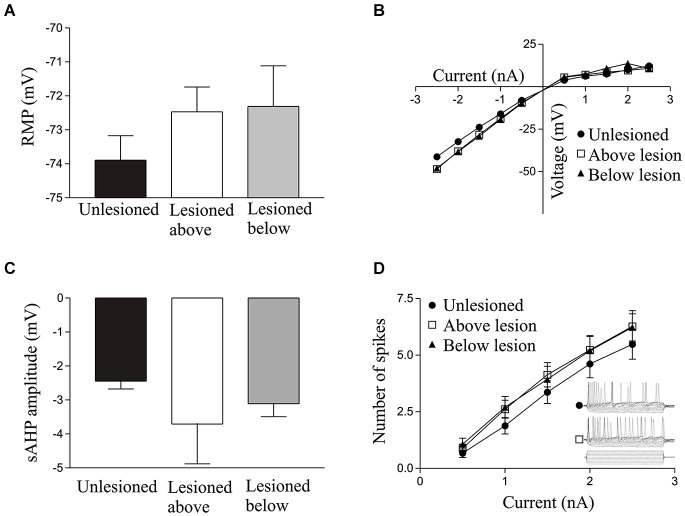
**Graphs showing values in unlesioned and lesioned animals for the resting membrane potential (A)**, I-V relationship **(B)**, slow afterhyperpolarisation (sAHP) amplitude **(C)**, excitability **(D)**; the inset shows sample 100 ms traces from cells in an unlesioned animal and a cell above the lesion site).

**Figure 2:**
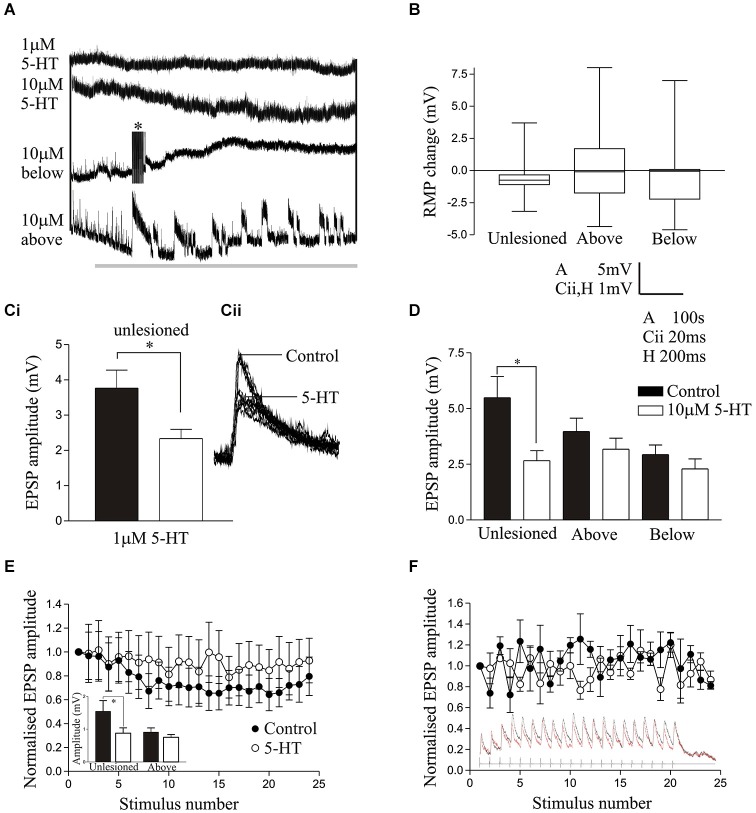
**(A)** The effects of 1 μM 5-HT and 10 μM 5-HT on the resting membrane potential of spinal cord neurons from unlesioned animals and lesioned animals above and below the lesion site. The asterix shows truncated spikes evoked by 5-HT. **(B)** Graph comparing the change in RMP by 10 μM 5-HT in unlesioned, above lesion and below lesion cells. **(Ci)** Graph and traces **(Cii)** showing the effect of 1 μM 5-HT on the cord stimulation-evoked EPSP amplitude. On this and other graphs * indicates statistically significant differences. **(D)** The effect of 10 μM 5-HT on the amplitude of cord-evoked EPSPs. Note that while there was a significant reduction in unlesioned animals, there was no significant effect above or below the lesion site. **(E)** The reticulospinal input over a spike train in an unlesioned animal in control and in 10 μM 5-HT. The inset graph shows the effect of 10 μM 5-HT on the initial EPSP in spike trains from an unlesioned and above lesion experiment. **(F)** Graph showing the lack of effect of 10 μM 5-HT on the activity-dependent plasticity of a reticulospinal input to a cell above the lesion site. The inset shows the synaptic input over the spike train.

**Figure 3:**
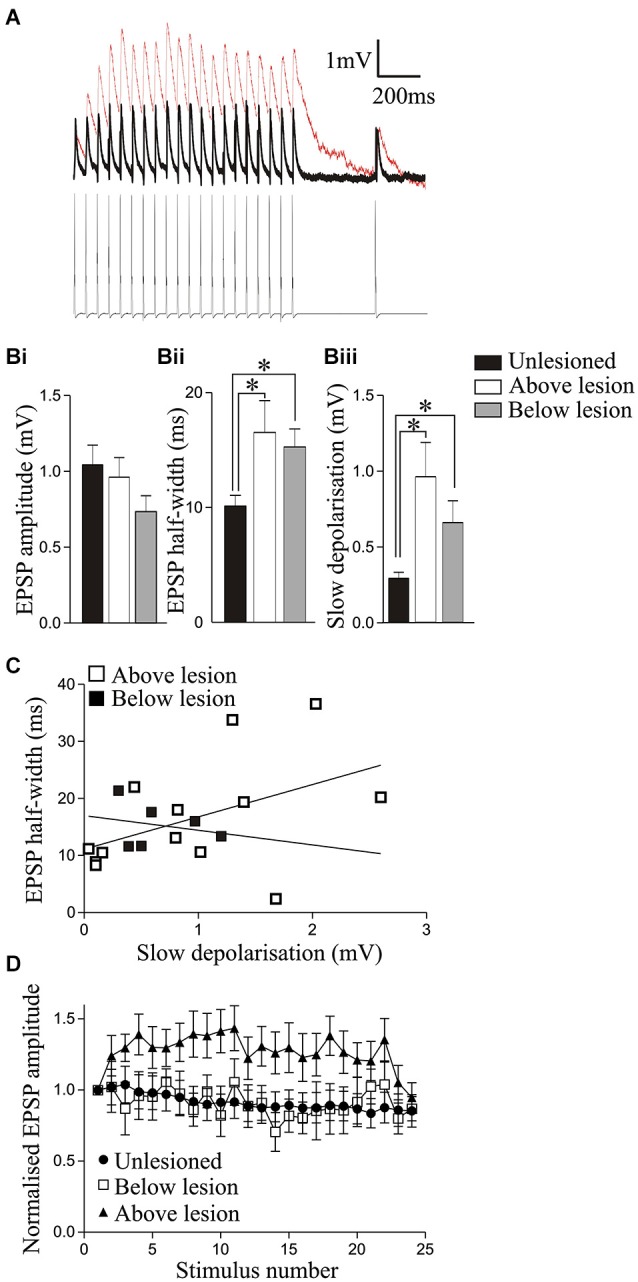
**Analysis of synaptic inputs from reticulospinal axons in unlesioned and lesioned animals above and below the lesion site. (A)** Traces showing the reticulospinal axon-evoked input in a cell recorded above the lesion site (red line) and in an unlesioned cord (black line). Note that the individual EPSPs in the lesioned cord sit on a slow depolarization. **(Bi)** The amplitude of the initial reticulospinal EPSP in spike trains did not differ significantly in unlesioned, above lesion, or below lesion cells, but the half-width **(Bii)** and the amplitude of the slow depolarization (measured from the pre-stimulation baseline to the baseline preceding the 20th EPSP); **(Biii)** were significantly greater above and below the lesion site compared to cells from unlesioned animals. **(C)** Graph showing the lack of correlation between the EPSP half-width and the slow synaptic depolarization amplitude above and below the lesion site. **(D)** Changes in the activity-dependent plasticity of reticulospinal inputs. Note that while inputs from unlesioned animals and below the lesion site depressed, the input above the lesion site facilitated. The *x* axis shows the stimuli number. The first 20 are successive stimuli all at 20 Hz; from 21–24 they are 250 ms, 550 ms, 2 s, and 4 s after the end of the 20 Hz train (see Section Methods).

**Figure 4:**
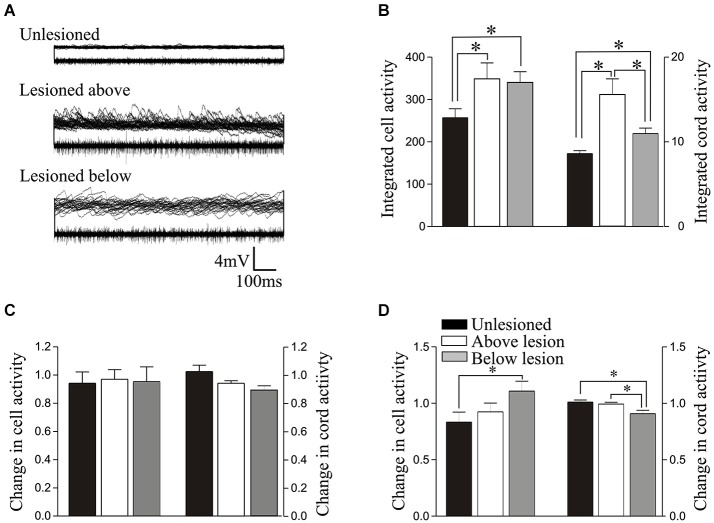
**(A)** Overlaid traces showing spontaneous synaptic activity recorded intracellularly (top of each of the paired trace) and activity recorded extracellularly from the surface of the spinal cord (bottom of each of the paired trace) from an unlesioned spinal cord, and from a lesioned spinal cord above and below the lesion site. **(B)** Graph showing the rectified and integrated cell and cord activity. **(C)** Graph showing the normalized change in integrated cord and cell activity by 1 μM 5-HT. **(D)** Comparison of the normalized changes in integrated cell and cord activity by 10 μM 5-HT. The key on D relates to all graphs on this figure.

**Figure 5:**
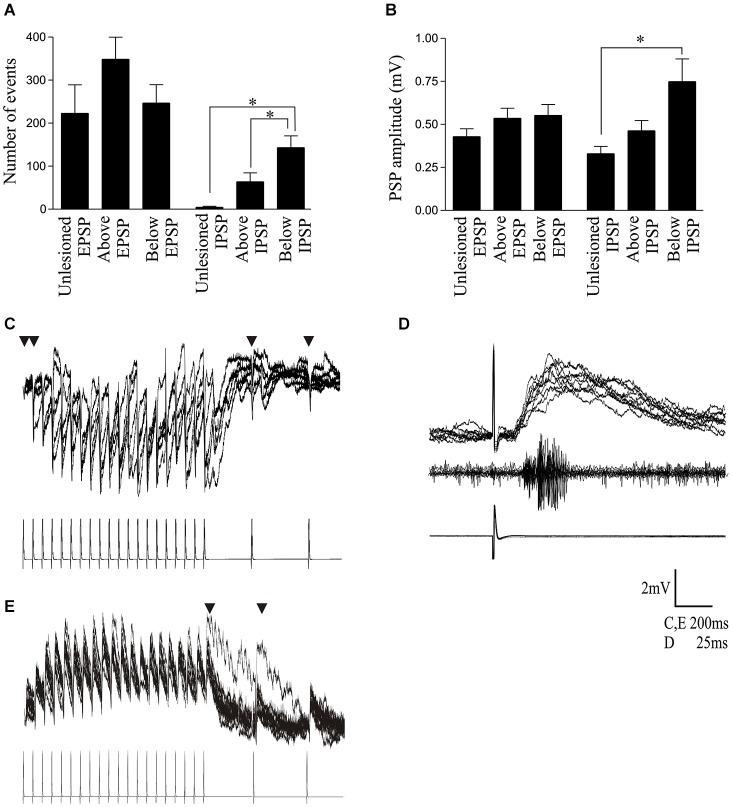
**(A)** Graph showing the number of spontaneous EPSPs and IPSPs in cells from unlesioned animals and above and below the lesion site. **(B)** Graph showing the spontaneous EPSP and IPSP amplitude. **(C)** Traces showing a polysynaptic IPSP evoked in a cell below the lesion site in response to 20 Hz stimulation of a reticulospinal axon. The inverted triangle indicates where presynaptic spikes failed to evoke a PSP on at least 50% of presynaptic stimulation trials. **(D)** Example of a polysynaptic EPSP evoked in a cell above the lesion site. Note the latency (~10 ms) before the response began and the multiple peaks on the depolarization. The middle trace shows a burst of activity recorded from the surface of the spinal cord approximately 5 segments below the cell, which also suggests activation of several cells by the single presynaptic spike. **(E)** Evidence of polysynaptic EPSP evoked by stimulating a reticulospinal axon that evoked a monosynaptic EPSP in a postsynaptic spinal cord neuron above the lesion site at 20 Hz. The inverted triangle indicates where polysynaptic EPSPs were evoked.

**Figure 6:**
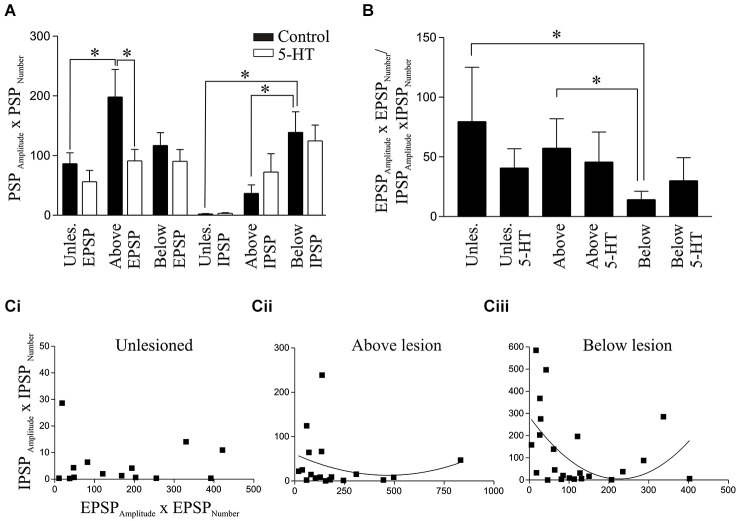
**Analysis of spontaneous PSPs. (A)** Graphs showing the product of the PSP amplitude and frequency for EPSPs and IPSPs in unlesioned and lesioned animals in control and in 1 μM 5-HT. **(B)** The ratio of the EPSP_Amplitude_ × EPSP_Number_ and IPSP_Amplitude_ × IPSP_Number_ in unlesioned animals and above and below the lesion site in control and in 1μM 5-HT. Note that the values were calculated for each cell and summed, and not from taking the averaged values for EPSPs and IPSPs from **(A)**. Correlation of the EPSP_Amplitude_ × EPSP_Number_ and IPSP_Amplitude_ × IPSP_Number_ in cells from unlesioned **(Ci)**, above lesion **(Cii)**, and below lesion spinal cords **(Ciii)**.

**Figure 7:**
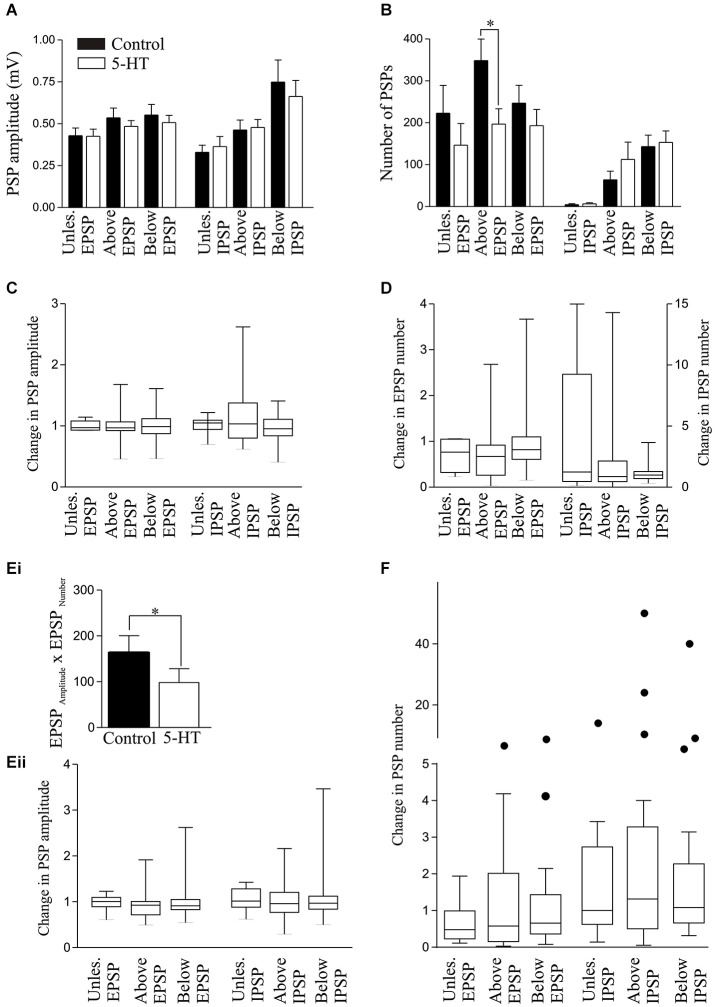
**Effect of 1 μM 5-HT on the spontaneous PSP amplitude (A) and number (B). (C)** Graph showing the normalized change in PSP amplitude in 5-HT. **(D)** Graph showing the normalized change in PSP number in 5-HT. **(Ei)** Graph showing the significant reduction of the EPSP_Amplitude_ × EPSP_Number_ in unlesioned animals by 10 μM 5-HT. **(Eii)** The effects of 5-HT on the normalized change in PSP amplitudes by 5-HT. **(F)** Graph showing the normalized change in the spontaneous PSP number in control and 10 μM 5-HT. The circles represent Tukey outliers, and highlight the increased variability in numbers after lesioning.

**Figure 8:**
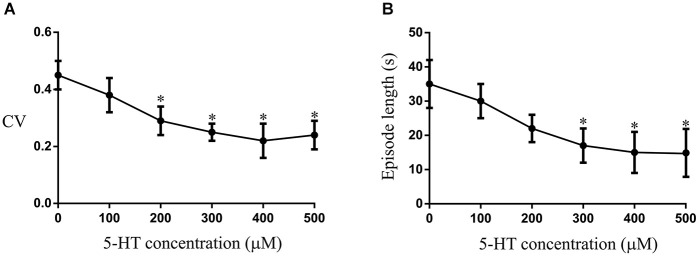
**The effects of different concentrations of 5-HT applied to the aquarium water on the CV(A) and episode length (B)**. The asterix shows that significant effects occurred at 200–300 μM, with a plateau effect occurring at 300 μM.

**Figure 9:**
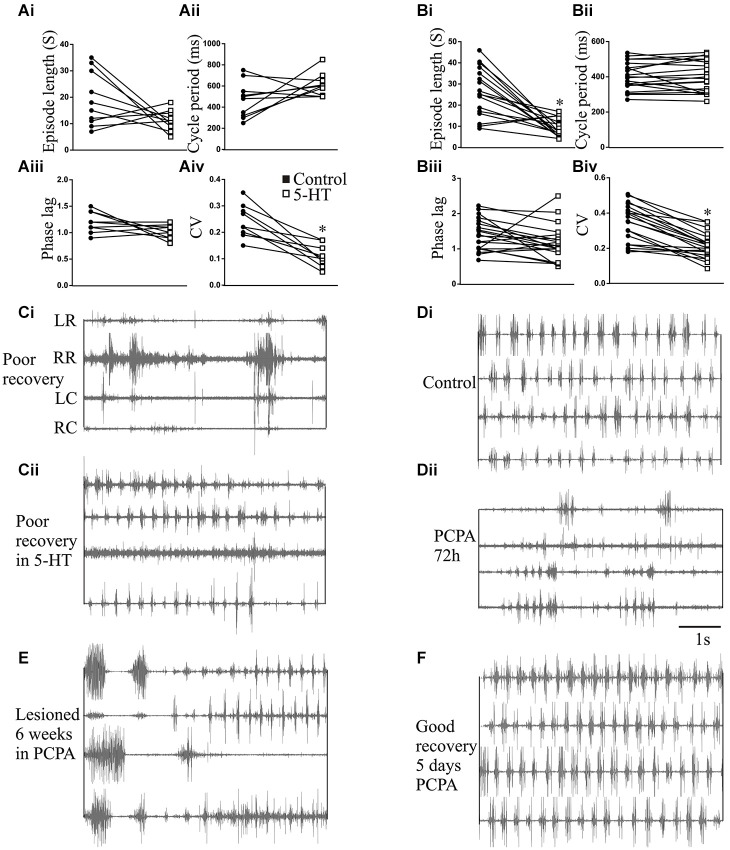
**The effects of 5-HT and 5-HT depletion on swimming**. Graphs showing the effects of 5-HT (500 μM) applied to the test chamber water on the episode length **(Ai)**, cycle period **(Aii)**, intersegmental phase lag **(Aiii)**, and burst coefficient of variation (CV; **Aiv**) in unlesioned animals, and the effects of 5-HT (500 μM) on the episode length **(Bi)**, cycle period **(Bii)**, intersegmental phase lag **(Biii)**, and burst coefficient of variation (CV; **Biv**) in lesioned animals. **(Ci)** EMG activity recorded in a poorly recovered animal and **(Cii)** the swimming in the same animal after 5-HT (500 μM) application. Electrode placement relative to the lesion site is shown to the left of the trace: LR, left rostral; RR, right rostral; LC, left caudal; RC, right caudal. **(Di)** EMG activity recorded in an unlesioned animal in control and **(Dii)** 72 h after incubation in PCPA to deplete 5-HT. **(E)** Traces showing disrupted swimming activity recorded 9 weeks after lesioning in an animal incubated in PCPA for the first six weeks after lesioning. **(F)** Lack of effect of 5 days incubation in PCPA in an animal that had recovered 10 weeks after a spinal cord lesion.

For intracellular and extracellular recordings animals were anesthetized with MS-222 and the spinal cord and notochord were removed from the trunk region (i.e., between the last gill and the start of the dorsal fin) in oxygenated lamprey Ringer at 4°C (Ringer contents: 138 mM NaCl, 2.1 mM KCl; 1.8 mM CaCl_2_; 2.6 mM MgCl_2_; 4.0 mM D-(+)-glucose; 2.0 mM HEPES; 0.5 mM L-glutamine, bubbled with O_2_ and adjusted to pH 7.4 with 1 M NaOH). The spinal cord was isolated from the notochord and pinned to a Sylgard lined chamber and superfused with lamprey Ringer at 10°C. Intracellular recordings were made from cells above and below the lesion site using an Axoclamp 2B amplifier (Molecular Devices). Motor neurons (identified by spikes in a ventral root following an evoked action potential in the cell body) and unidentified cells were examined in unlesioned animals and in lesioned animals above and below the lesion site. Given the relative size and number of different types of neurons, unidentified cells are likely to be motor neurons: as there were no obvious differences in motor neurons and unidentified cells they were grouped for analysis (identified motor neurons constituted at least 80% of the sample size in each analysis). Recordings were not made from different classes of interneurons as this analysis is not trivial even in this “simpler” system (Parker, 2006, 2010) and we initially need to identify the changes that invite targeted cell and synapse-specific analyses. While the analysis essentially treats the locomotor network as a functional unit, using motor neurons allowed us to assay cellular properties, while also inferring something about premotor inputs from spontaneous synaptic activity. Cells were typically sought 2–3 segments rostral or caudal to the lesion site. The resting membrane potential (RMP) was examined after the cell had stabilized (~2 min); the input resistance was measured by injecting 100 ms hyperpolarising current pulses (−0.5 to −2.5 nA) into cells under discontinuous current clamp (DCC; sampling frequency between 2–3 KHz); excitability was examined by injecting 100 ms depolarizing current pulses (0.5–2.5 nA) into the cells under DCC.

Spontaneous spinal cord activity was recorded extracellularly by placing a glass suction electrode on the surface of the spinal cord to cover the cell body area, and intracellularly by recording spontaneous synaptic activity from a cell in thirty 1 s sweeps. Cord and intracellular activity was rectified and integrated in Clampfit to quantify the summed activity. As the cellular activity was rectified it reflected the total changes in synaptic input from premotor neurons and associated changes in resting potential and thus provided a measure of the total subthreshold activity of the cell. To examine EPSP and IPSP properties specifically (albeit from unidentified presynaptic sources) spontaneous PSPs were instead measured over a 40 s period in each cell using the event detection feature in Clampfit.

The properties of specific presynaptic inputs were examined by making paired intracellular recordings from postsynaptic motor neurons and Müller reticulospinal axons. These inputs were also used to examine the modulatory effects of 5-HT, but for this analysis cord stimulation-evoked responses were also used when reticulospinal axons that connected to the recorded postsynaptic cell could not be found. Cord-stimulation responses were evoked by stimulating the medial column that runs between the cell body layer (the region where locomotor network interneurons and motor neurons are located) and the midline of the spinal cord extracellularly using a glass suction electrode for 1 ms at 0.1 Hz for 50 s. The stimulation strength was adjusted to evoke what appeared to be a unitary EPSP, determined by the short rise time, longer decay time, and absence of multiple peaks (however, with any extracellular stimulation the potential exists for multiple inputs being evoked onto the postsynaptic cell). This was done in control and in the presence of 5-HT: the overlaid PSPs were averaged and the peak amplitude was measured. Putative monosynaptic reticulospinal-evoked inputs were examined by intracellular stimulation of reticulospinal axons in the medial column above the lesion site several segments rostral to the postsynaptic cell at 20 Hz for 1 s, followed by low frequency recovery test pulses (250 ms, 550 ms, 2 s, and 4 s after the end of the train). The amplitude of the initial EPSP and of EPSPs over the spike train was measured from the baseline preceding each EPSP to the peak of the EPSP. The trains were evoked at 20 s intervals. As there is no activity-dependent plasticity of the input at this frequency, the initial EPSP in the spike trains provided a measure of single low-frequency-evoked EPSPs. Putative monosynaptic connections were determined by the presence of reliable EPSPs that occurred with a consistent short latency (typically <2–3 ms) in response to presynaptic stimulation at 20 Hz (note that functionally weak connections (e.g., regenerated axons) could fail the criteria for monosynapticity even though the connections were monosynaptic). Inputs were considered to be putatively polysynaptic if the inputs were unreliable (i.e., presynaptic spikes failed to evoke a PSP; see Figure 5C), had a relatively long latency to the postsynaptic response (10 ms or greater; see Figure 5D), and had multiple peaks on the postsynaptic depolarization (see Figures 5D,E). The unequivocal identification of monosynaptic or polysynaptic inputs is not trivial (Berry and Pentreath, 1976), and the use of the term putative relates to the likelihood that an input is mono- or polysynaptic.

Drugs were purchased from Sigma-Aldrich. Drugs were applied to the isolated spinal cord by superfusion using a peristaltic pump. The data presented here are taken from two batches of animals examined over a two year period, and does not include data from previous analyses. Statistical analyses were performed in Graphpad Prism using Wilcoxon matched pairs test for paired comparisons or a Kruskal-Wallis test with a *post hoc* Dunns test for multiple comparisons. The graphs show mean responses ± SEM.

## Results

### Effects of 5-HT in unlesioned and lesioned animals

We examined if there were changes in modulatory effects after spinal cord lesions by comparing the effects of 5-HT in the isolated spinal cord of lesioned and unlesioned animals. As we have used juvenile adults rather than larvae for the first time in our analyses, we have also compared cellular and synaptic properties in lesioned and unlesioned spinal cords (see Cooke and Parker, 2009 for analyses in larvae).

There were no significant differences in unlesioned and lesioned animals (either above or below the lesion site) in the resting potential (RMP; Figure 1A), input resistance (Figure 1B), I-V relationship (Figure 1B), the slow afterhyperpolarisation (sAHP) amplitude (Figure 1C), or the excitability (Figure 1D; see Table 1).

**Table 1:** **Comparison of cellular properties in unlesioned and lesioned animals**.

	RMP	Input resistance	sAHP	Cord integrated	Cell integrated
Unlesioned	−73.9 ± 0.7 mV (*n* = 74)	16.2 ± 1.7 MΩ (*n* = 59)	−2.4 ± 0.2 mV (*n* = 59)	8.6 ± 0.3 (*n* = 42)	257 ± 22 (*n* = 42)
Above lesion	−72.5 ± 0.7 mV (*n* = 42)	19.4 ± 1.7 MΩ (*n* = 32)	−3.7 ± 1.2 mV (*n* = 32)	15.6 ± 1.9^*^# (*n* = 42)	349 ± 38^*^ (*n* = 42)
Below lesion	−72.3 ± 1.2 mV (*n* = 81)	19.4 ± 2.3 MΩ (*n* = 66)	−3.1 ± 0.04 mV (*n* = 66)	11 ± 0.7^*^ (*n* = 66)	340 ± 25^*^ (*n* = 66)

In cells from unlesioned animals, 1 μM 5-HT non-significantly hyperpolarised the RMP (−1.16 ± 0.5 mV, *n* = 6; Figure 2A), an effect that did not differ significantly to that in lesioned animals above (−0.95 ± 0.5 mV, *n* = 19) or below the lesion site (−1.3 ± 0.6 mV, *n* = 22; *p* > 0.05; data not shown). With 10 μM 5-HT (Harris-Warrick and Cohen, 1985) there was again a consistent, but non-significant, hyperpolarization of the membrane potential in unlesioned animals (*n* = 15 of 16; −0.61 ± 0.34 mV; Figure 2A). However, below the lesion site it depolarized the membrane potential in the majority of cells (*n* = 16 of 30 cells), but in the population as a whole there was still a mean hyperpolarization of −0.54 ± 0.39 mV, while above the lesion site it depolarized the membrane potential in 9 of 24 cells, which in this case gave a mean depolarization of 0.19 ± 0.76 mV (Figure 2A). While the mean RMP changes by 10 μM 5-HT were not significantly different (Figure 2B), there was a significant increase in the proportion of cells that depolarized above and below the lesion site compared to unlesioned animals (*p* < 0.05, Chi square). The RMP effect thus changed from a highly consistent hyperpolarization in unlesioned animals to a more variable effect where there was a mix of depolarization and hyperpolarization.

There was no significant effects of 1 μM 5-HT on the input resistance, I-V relationship, excitability, or sAHP amplitude in unlesioned animals or lesioned animals above or below the lesion site (data not shown). For 10 μM 5-HT there was again no significant effect on the excitability or I-V relationship in cells from unlesioned animals or lesioned animals above or below the lesion site (*p* > 0.05; data not shown), but it did significantly reduce the sAHP amplitude in each case (unlesioned (*n* = 11), above lesion (*n* = 10), lesioned below (*n* = 25), the reduction not differing significantly in the different conditions (*p* > 0.05; data not shown).

Cord-stimulation-evoked EPSPs were significantly reduced in spinal neurons by 1 μM 5-HT in unlesioned animals (Figures 2Ci,ii; *p* < 0.05, *n* = 6), and in lesioned animals above (*n* = 19) and below the lesion site (*n* = 20), the magnitude of the EPSP reduction not differing significantly between conditions (*p* > 0.05; data not shown). Similarly, 10 μM 5-HT also significantly reduced the amplitude of cord stimulation-evoked EPSPs in unlesioned animals (*n* = 11; *p* < 0.05; Figure 2D). However, it had no overall significant effect on the EPSP amplitude above (*n* = 21) or below (*n* = 26) the lesion site (*p* > 0.05; Figure 2D), suggesting a weakening of 5-HT synaptic effects after lesioning. The effects of 10 μM 5-HT were also examined on reticulospinal-evoked EPSPs in spinal neurons in unlesioned animals (*n* = 11) and in lesioned animals above the lesion site (*n* = 3): it is generally harder to find connections in lesioned animals, especially below the lesion site, which may be a reflection of the sparseness of regenerated inputs (Oliphint et al., 2010). The sample of above lesion connections is too small for a statistical analysis and only shows potential trends. There was a significant reduction of the initial EPSP amplitude in the spike train by 10 μM 5-HT, but as with cord stimulation-evoked EPSP there was no change in the EPSP amplitude above the lesion site (inset Figure 2E). 5-HT non-significantly reduced depression in unlesioned animals (Figure 2E), possibly due to the reduction of the initial EPSP amplitude, but there was no 5-HT change in the properties of the EPSP in reticulospinal axons above the lesion site (Figure 2F).

Reticulospinal-evoked EPSPs examined in the isolated spinal cord were used to examine lesion-induced differences in the basic properties of evoked synaptic inputs. There was no significant difference in the initial EPSP amplitude in the spike train in unlesioned or lesioned animals above or below the lesion site (Table 2; Figures 3A,Bi). The EPSP rise time did not differ, but the half width was significantly greater above and below the lesion site than in unlesioned animals (Figure 3Bii; Table 2). This could reflect potentiation of the NMDA component of the EPSP, which correlates with the EPSP half-width (Dale and Grillner, 1986). The slow synaptic depolarization over spike trains seen in lesioned larval animals (Cooke and Parker, 2009) was present (Figure 3A). This effect was significantly greater in lesioned than in unlesioned animals, but it did not differ significantly above and below the lesion site (Figures 3A,Biii; Table 2). The mechanisms underlying this effect are unknown: it is not blocked by high calcium Ringer (data not shown), suggesting either a monosynaptic or a strong polysynaptic effect (see Berry and Pentreath, 1976). It was probably not due to the increased EPSP half-width, as there was no significant correlation between the slow depolarization amplitude and the half-width above or below the lesion (Figure 3C; *r*^2^ = 0.21 above, *r*^2^ = 0.06 below, *p* > 0.05). In unlesioned animals and lesioned animals below the lesion site when all connections were averaged the EPSP depressed across the spike train (Figure 3D). However, above the lesion site the input significantly facilitated (*p* < 0.05; Figures 3A,D). This facilitation from an unchanged initial EPSP amplitude will make connections above the lesion functionally stronger, and suggests a functional difference above and below the lesion site.

**Table 2:** **Comparison of reticulospinal-evoked ESPP amplitudes in unlesioned and lesioned animals**.

	RS EPSP amplitude	RS EPSP rise time	RS EPSP half-width	Slow depolarization	Train
Unlesioned	1.12 ± 0.11 mV (*n* = 47)	2.8 ± 0.7 ms (*n* = 17)	10.1 ± 0.9 ms (*n* = 17)	0.29 ± 0.04 mV (*n* = 24)	0.87 ± 0.1 (*n* = 24)
Above lesion	0.96 ± 0.13 mV (*n* = 13)	2.5 ± 0.7 ms (*n* = 13)	15.5 ± 9.9 ms (n = 13)*	0.96 ± 1.8 mV (*n* = 13)*	0.92 ± 0.2 (*n* = 13)
Below lesion	0.73 ± 0.1 mV (*n* = 6)	2.9 ± 0.1.1 ms (*n* = 6)	15.3 ± 3.8 ms (*n* = 6)*	0.76 ± 0.8 mV (*n* = 6)*	1.2 ± 0.12 (*n* = 6)*

The integrated synaptic activity (see Section Methods) was significantly increased in cells above and below the lesion site compared to cells from unlesioned animals (Figures 4A,B; Table 1). The integrated spontaneous activity recorded extracellularly from the surface of the cord was also significantly increased above and below the lesion site compared to unlesioned animals. In this case activity above the lesion site was also significantly greater than activity below (Figures 4A,B; Table 1), which again suggests a functional difference on either side of the lesion site. There was no significant difference in the effect of 1 μM 5-HT on integrated synaptic or extracellular activity (*p* > 0.05; Figure 4C). However, there was a significant increase in integrated synaptic activity in 10 μM 5-HT below (*p* < 0.05, *n* = 22), but not above (*n* = 15), the lesion site compared to unlesioned animals (*n* = 9; Figure 4D), and a significant decrease in the integrated extracellular activity below compared to both unlesioned and above lesion values (*p* < 0.05; Figure 4D).

As the integrated synaptic activity reflects the total spontaneous synaptic input, discrete spontaneous EPSPs and IPSPs were measured to examine this activity further. There were no significant differences in the amplitude or number of spontaneous EPSPs in cells from unlesioned animals or lesioned animals above and below the lesion site (Figures 5A,B; Table 3). However, the number and amplitude of IPSPs was significantly greater below the lesion site than in unlesioned animals, and the number of IPSPs was also significantly greater below than above the lesion (Figures 5A,B; Table 3). This relative increase in inhibition could contribute to the reduced integrated cord activity below the lesion site (Figure 4B), and the change in integrated spontaneous synaptic activity (Figures 4A,B). The increase in inhibition was supported by the increase in the proportion of putative polysynaptic IPSPs (see Section Methods) seen in paired recordings from reticulospinal axons and motor neurons below the lesion site (Figure 5C; *n* = 1 of 17 in unlesioned animals, *n* = 3 of 6 lesioned animals, *p* < 0.05, Chi square). While the properties of spontaneous PSPs above the lesion site did not differ to unlesioned animals, there was additional evidence for an increase in excitatory connectivity: putative polysynaptic EPSPs were more common above the lesion site than in unlesioned animals (*n* = 1 of 47 in unlesioned compared to 7 of 13 in lesioned animals, *p* < 0.05, Chi square; see Figures 5D,E). This suggests a strengthening of evoked feedforward excitatory connections despite the lack of change of spontaneous inputs (see Goel and Buonomano, 2013).

**Table 3:** **Characterization of spontaneous PSPs in unlesioned and lesioned animals**.

	Spontaneous EPSP Amp	Spontaneous EPSP No.	Spontaneous IPSP Amp	Spontaneous IPSP No.
Unlesioned	0.46 ± 0.03 mV (*n* = 34)	215 ± 38 (*n* = 34)	0.32 ± 0.04 mV (*n* = 33)	7 ± 18 (*n* = 33)
Above lesion	0.53 ± 0.06 mV (*n* = 19)	348 ± 51 (*n* = 19)	0.46 ± 0.12 mV (*n* = 18)	68 ± 21 (*n* = 18)
Below lesion	0.51 ± 0.03 mV (*n* = 24)	229 ± 40 (*n* = 24)	0.67 ± 0.04 mV (*n* = 23)*	145 ± 28 (*n* = 23)*#

An estimate of the summed spontaneous excitatory or inhibitory drive for each cell was quantified from the product of the PSP amplitude and number (PSP_Amplitude_ × PSP_Number_). The IPSP value for spinal cord injured animals was significantly greater below than those in both the unlesioned and above lesion values, while the EPSP value was significantly greater above than in unlesioned animals (Figure 6A). When the EPSP:IPSP ratio (EPSP_Amplitude_ × EPSP_Number_ /IPSP_Amplitude_ × IPSP_Number_) was calculated for each cell there was a significant reduction below the lesion site compared to unlesioned and above lesion cases, presumably reflecting the increase in inhibition (Figure 6B). This relationship was examined further by correlating the IPSP_Amplitude_ × IPSP_Number_ against the EPSP_Amlitude_ × EPSP_Number_. This analysis showed little relationship in unlesioned animals where there was a similar inhibitory value over a range of excitatory values (Figure 6Ci). Below the lesion site the IPSP_Amplitude_ × IPSP_Number_ was larger with smaller and larger excitatory values, possibly reflecting the increased feedforward activation of inhibitory interneurons (see Figure 5C): at intermediate levels of excitation IPSP values were low, giving a parabolic relationship. This was only significant below the lesion (below *r*^2^ = 0.25, *p* < 0.05; above lesion *r*^2^ = 0.04, *p* > 0.05; (Figures 6Ci–iii), and suggests a reorganization of the spinal cord circuitry that reflects a need for greater inhibition below the lesion site as excitation is increased.

There was no significant effect of 1 μM 5-HT on the spontaneous EPSP or IPSP amplitude in cells from unlesioned animals or cells above or below the lesion site (*p* > 0.05, *n* = 6; Figure 7A). However, it significantly reduced the number of EPSPs and the EPSP_Amplitude_ × EPSP_Number_ above the lesion site (*p* < 0.05; Figures 6A, 7B), suggesting a lesion-induced change in 5-HT sensitivity at this site. The analysis of 5-HT effects on spontaneous PSPs was complicated by two features: firstly there was marked variability in initial values, especially for the number of PSPs (range from <10 to >600); and secondly, 5-HT could evoke oscillations of the membrane potential that could be associated with phases of increased and decreased spontaneous inputs (see Figure 2A). These oscillations are difficult to control for here: effects were measured 10 min after 5-HT application and varying this to select a region where there was or was not an oscillation would bias the analysis. The variability of initial values could, however, be addressed by normalizing the control values and analyzing the change in 5-HT (Figures 7C,D). In this case there were again no significant differences, but the greater variability after lesioning can be seen from the increased ranges when the data is shown on box-plots. 10 μM 5-HT also failed to significantly affect the spontaneous IPSP or EPSP amplitude or number, but it significantly reduced the EPSP_Amplitude_ × EPSP_Number_ in unlesioned animals (Figure 7Ei). Variability was again a feature, especially for the number of events. However, no significant effects were revealed by normalizing the change in 5-HT to the pre-5-HT value (Figures 7Eii,F).

In summary, these results show significant differences in cellular and synaptic properties and their modulation by 5HT in the isolated lesioned spinal cords, and that these changes differ above and below the lesion site.

### 5-HT effects on swimming

The changes in the modulatory effects of 5-HT in lesioned animals obviously need to be understood in the context of their role in functional recovery. This is difficult to address as it requires direct links between cellular/synaptic effects and behavior, something that is far from trivial even in this simpler system (see Parker, 2006, 2010). To provide a basis for these analyses we need to know the behavioral effects of 5-HT. This was examined in intact swimming lampreys. In unlesioned animals (*n* = 10) significant effects of 5-HT on the CV and episode length occurred at concentrations of between 200–300 μM (Figures 8A,B). The effects of 500 μM 5-HT are presented in Figure 9. 5-HT had variable non-significant effects on the swimming episode length (*p* > 0.05; Figure 9Ai; all values on this figure are 500 μM). Similar effects were seen for the cycle period. Overall there was no significant change (*p* > 0.05), but control values varied and in animals with shorter cycle periods (<400 ms) the cycle period was increased (Figure 9Aii). There was no significant effect on the phase lag (*p* > 0.05; Figure 9Aiii), but the variability of swimming was reduced by 5-HT, shown by the significant reduction of the coefficient of variation (CV; *p* < 0.05; Figure 9Aiv).

In lesioned animals (*n* = 20), the swimming episode length was significantly reduced by 5-HT at higher concentrations (400–500 μM; *p* < 0.05; Figure 9Bi), although the effect was again greater in animals that showed longer swimming episodes in control. In these animals, there was no significant overall or apparent state-dependent effect on the cycle period (Figure 9Bii) or the phase lag (Figure 9Biii; *p* > 0.05), but the CV was again significantly reduced (*p* < 0.05; Figure 9Biv).

While the *n* number is low (*n* = 3), 5-HT could improve swimming in poorly recovered animals (swimming score of 2). In these animals activity was absent below the lesion site but was evoked by 5-HT, and the activity above the lesion site became more regular (Figure 9C). These effects resulted in an increase in the swimming score from 2 to 3/4, and so swimming was still far from fully recovered.

The role of 5-HT in promoting recovery was examined using PCPA to deplete 5-HT (Hashimoto and Fukuda, 1991; Airhart et al., 2012). PCPA did not cause any acute effects (tested from 1–24 h after incubation; data not shown), but incubating unlesioned animals in PCPA for 72 h markedly disrupted locomotor activity to an extent that locomotor parameters could not be measured for a quantitative analysis. This suggests a necessary role for 5-HT in normal swimming (Figures 9Di,ii). On removal from PCPA the animals regained good locomotor function within 3–5 days, to an extent that the episode length, cycle period, intersegmental phase lag, and CV did not differ significantly to non-exposed control animals (data not shown). Incubating lesioned animals in PCPA for 6 weeks resulted in poor recovery in 5 of 5 animals (all assessed as stage 2 or 3; Figure 9E). A matched group of control lesioned animals that were not incubated in PCPA all recovered good function (stage 5 or 6; *p* < 0.05 Chi square). The failure of PCPA incubated animals to recover was not due to the acute effect of PCPA as animals were tested after being removed from PCPA for at least 2 weeks. However, in the five animals that recovered in the absence of PCPA, incubation in PCPA for 5 days 10 weeks after lesioning resulted in no disruption of locomotor activity (Figure 9F). This contrasts the effect in unlesioned animals and suggests that while 5-HT is necessary for swimming in unlesioned animals and for recovery after lesioning, it is not needed to maintain recovery.

### Relationship to degree of recovery

We have previously found significant differences in larval animals separated into those that recovered well or poorly (see Cooke and Parker, 2009; Hoffman and Parker, 2011). We thus separated lesioned animals into those that recovered well or poorly by taking the upper (stage 5 or 6, *n* = 39) and lower levels of the swimming score (stage 2 or 3, *n* = 10).

**Figure 10:**
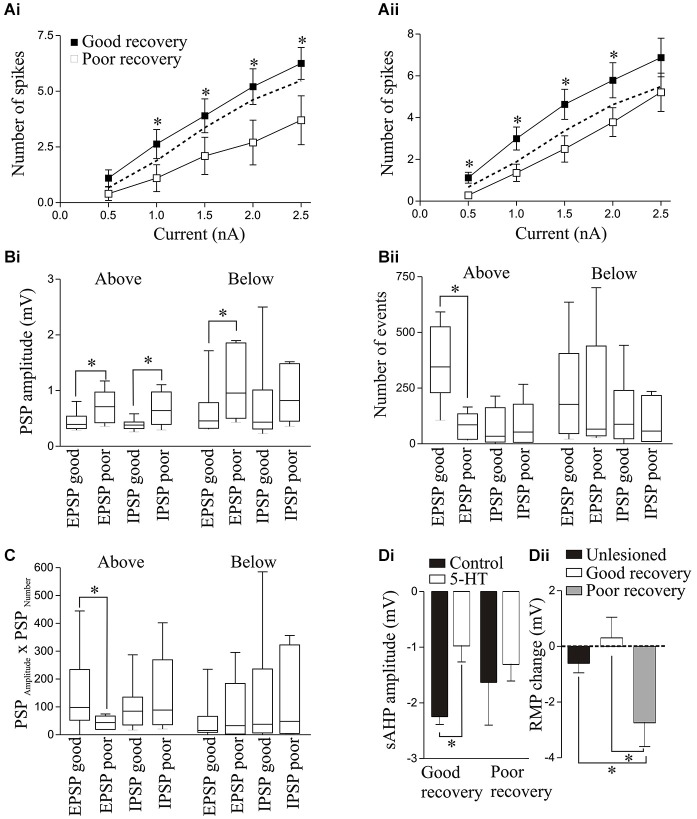
**Excitability in response to 100ms current pulse steps in animals that recovered well or poorly above (Ai) and below (Aii) the lesion site**. The dashed line shows the unlesioned response. The spontaneous PSP amplitude **(Bi)** and number **(Bii)**, and the PSP_Amplitude_ × PSP_Number_ **(C)** above and below the lesion site in animals that recovered well or poorly. **(Di)** Graph showing the significant reduction in the sAHP amplitude by 5-HT (10 μM) T (10HT (above the lesion site in animals that recovered well but not in those that recovered poorly. **(Dii)** Graph showing the change in resting membrane potential (RMP) by 5-HT below the lesion site in unlesioned animals and animals that recovered well or poorly.

There was no significant difference in RMP, input resistance, or the sAHP amplitude above or below the lesion site in the isolated spinal cord from animals that recovered well or poorly (data not shown). Excitability did not differ to that in unlesioned animals for either those that recovered well (*n* = 20) or poorly (*n* = 10), but above the lesion site excitability was significantly reduced with current steps from 1–2.5 nA in poorly recovered animals (Figure 10Ai). A similar effect occurred below the lesion site, but here excitability in response to 0.5–2 nA current steps was significantly lower in poorly recovered animals (*n* = 14) than those that recovered well (*n* = 33; Figure 10Aii).

There was a significant increase in spontaneous EPSP and IPSP amplitudes above the lesion site in the isolated spinal cord from poorly recovered animals (*n* = 6) compared to those that recovered well (*n* = 10; Figure 10Bi), while the number of EPSPs was significantly less in poorly recovered animals (Figure 10Bii). The spontaneous EPSP amplitude was also significantly greater below the lesion site in poorly recovered animals (*n* = 6) compared to those that recovered well (*n* = 17). The EPSP_Amplitude_ × EPSP_Number_ was significantly greater above the lesion site in animals that recovered well than in those that recovered poorly (Figure 10C), an effect that was consistent with an increase in the integrated synaptic activity above the lesion site in animals that recovered well (data not shown): no other values differed significantly.

For 5-HT (10 μM) effects, above the lesion site the only significant difference was that the sAHP was only significantly reduced in animals that recovered well (Figure 10Di). Below the lesion site the 5-HT effect on the RMP was significantly different. In unlesioned animals 10 μM 5-HT non-significantly hyperpolarised the membrane potential (*n* = 15 of 16, mean effect −0.61 ± 0.34 mV; Figure 2A). In good recovery there were variable effects on the RMP (*n* = 3 depolarize, *n* = 4 hyperpolarize, *n* = 5 unchanged) that gave a mean change of 0.31 ± 0.74 mV (*n* = 12), but in cells from poorly recovered there was a hyperpolarization in every cell that was significantly different to cells from animals that recovered well (−2.7 ± 0.86 mV; *p* < 0.05, *n* = 10; Figure 10Dii).

## Discussion

While several of the effects shown here are preliminary and require further analysis, the results show that there are changes in cellular and synaptic properties in the isolated lesioned compared to the isolated unlesioned spinal cord; that these changes differ above and below the lesion site; and that the modulatory effects of 5-HT are altered by lesioning, with effects again differing above and below the lesion site.

There were several changes in functional properties in lesioned animals. Spontaneous synaptic and cord activity was increased above and below the lesion site compared to unlesioned animals. The reticulospinal axon-evoked EPSP half-width and the slow synaptic depolarization during trains of action potentials were also greater above and below the lesion site compared to unlesioned animals. However, the activity-dependent plasticity of reticulospinal inputs differed above and below the lesion site: depression occurred below that matched that seen in unlesioned animals, but above the lesion site the input facilitated which will make these connections functionally stronger. Facilitation is typically associated with a lower release probability and smaller initial EPSP amplitude (Zucker and Regehr, 2002). The size of the active zone can correlate with the release probability (see Holderith et al., 2012 and references therein), and the active zone is reduced at regenerated larval lamprey Müller reticulospinal axons (Oliphint et al., 2010). However, there was no reduction of the initial EPSP amplitude which would be expected if the release probability was reduced. A reduction of release probability to allow facilitation without a reduction of the initial EPSP amplitude is possible if the number of available vesicles is increased (Bevan and Parker, 2004), but this possibility requires further analysis, as do the general properties of synapses made by regenerated axons. For example, while the mean amplitude of monosynaptic EPSPs does not differ in unlesioned and lesioned animals, regenerated Müller axons make fewer synaptic contacts below the lesion site (Oliphint et al., 2010). The generation of an EPSP of comparable amplitude to that in unlesioned animals from a smaller number of synaptic contacts should require some change in the release properties or postsynaptic effects of the individual contacts.

The EPSP_Amplitude_ × EPSP_Number_ was greater above the lesion site than in unlesioned animals, suggesting potentiation of the excitatory drive. This could relate to the increased incidence of putative polysynaptic EPSPs above the lesion site: this is consistent with greater feedforward excitation, and could reflect potentiation of connections onto or between the excitatory interneurons (EIN), changes in EIN excitability (Parker, 2003), or strengthening of crossing excitatory connections (ScIN or CCIN; Buchanan, 2001; Parker, 2003). Conversely, putative polysynaptic IPSPs, the spontaneous IPSP amplitude and number, and the IPSP_Amplitude_ × IPSP_Number_ were greater below the lesion site. This suggests changes in inhibitory premotor interneuron excitability or synaptic strengths (this could either occur ipsilaterally (SiIN or LIN; Buchanan, 2001; Parker, 2003) or contralaterally (ScIN or CCIN; Buchanan, 2001; Parker, 2006)) or a change in the excitatory drive to these cells. The latter effect is supported by the parabolic relationship between the EPSP_Amplitude_ × EPSP_Number_ and IPSP_Amplitude_ × IPSP_Number_. These region specific changes in inhibition and excitation now suggest specific network synaptic interactions that can be targeted for analysis above and below the lesion site.

Here we have used juvenile adults for the first time in our analyses rather than larvae (Cooke and Parker, 2009; Hoffman and Parker, 2011). Several effects differed here to those in larvae. This could reflect developmental influences (Parker and Gilbey, 2007; Cooke et al., 2012), not a surprising conclusion but one that merits consideration to avoid the mixing of developmental stages. In contrast to larvae (Cooke and Parker, 2009), there were no significant changes in cellular properties (resting potential, input resistance, sAHP amplitude, or excitability). This suggests a potential developmental switch from changes in cellular to changes in synaptic properties. Here inhibition was also greater below the lesion site, but in larvae inhibition was only increased in poorly recovered animals. However, as noted above this increased inhibition could reflect an increase in feedforward excitation.

We have also examined changes above the lesion site for the first time. That there are changes here argues against lesion-induced differences simply being a consequence of the removal of descending regulatory inputs (which has been considered to explain spasticity below lesion sites; Dietz, 2002) or a compensatory response to the removal of descending excitation (Cooke and Parker, 2009). The above lesion effects may instead reflect the need for changes at multiple sites that act together to regulate the integrated activity of the supra and sub-lesion spinal cord to generate an efficient motor output. Increased activity above the lesion site may also be needed to drive activity below, either neuronally through increased excitation of regenerated propriospinal axons, or mechanically by strong supra-lesional movements that propagate below the lesion site to be relayed to the spinal cord by potentiated proprioceptive inputs (Hoffman and Parker, 2011). Grasso et al. (2004) provided evidence of mechanical effects after injury to the human spinal cord (e.g., using arm and body movements to assist leg movement), and Shah et al. (2013) showed that forelimb training increased hindlimb function in rats, suggesting that mechanical propagation of this sort is not simply a peculiarity of the lamprey. In contrast to many species, ascending inputs also regenerate in lamprey (Armstrong et al., 2003), and the reduction or loss of these ascending inputs could provide a signal to cells above the lesion site that drive the supra-lesion changes in functional properties.

### Lesion-induced changes in the effects of 5-HT

A principal focus of the analysis was to compare the effects of 5-HT in lesioned and unlesioned animals. The potential role for neuromodulators, especially 5-HT, in restoring function after SPI has been a major focus of research (see Rossignol et al., 2001). However, little is known if or how injury influences modulatory effects. Changes in modulation could occur directly through changes in the properties of transmitter receptors or second messenger pathways, indirectly through altered interactions between modulatory systems as a result of the loss or reduction of one or more transmitter systems, or through state-dependent effects caused by the lesion-induced changes in cellular and synaptic properties. The different effects of 5-HT in unlesioned and lesioned animals shown here add to the evidence of changes in modulatory effects after spinal cord lesions (Svensson et al., 2013). In unlesioned animals, 5-HT had effects that should generally reduced excitation, shown by the consistent, albeit non-significant, hyperpolarization of the membrane potential and the reduction of evoked EPSP amplitudes and spontaneous EPSPs, but in lesioned animals both of these effects were absent. The differences in 5-HT effects after lesioning suggest that pharmacological approaches to functional recovery should not be assumed from analyses in unlesioned spinal cords. There were also differences in 5-HT effects above and below the lesion site. As systemic drug application will act at both sites it may be beneficial to target potential regional effects. This would be possible if effects are mediated by different receptor subtypes.

The behavioral analyses of 5-HT effects on swimming provide preliminary evidence of a role for 5-HT in recovery. While there are several caveats to the analysis of swimming, including unknown drug concentrations in the CNS and uncertainty of their sites of action, a consistent result in these experiments was that putative 5-HT depletion using PCPA led to a failure of recovery (note however that we have not measured 5-HT levels after this treatment). Exogenous and endogenous 5-HT slows the frequency of network activity in unlesioned animals (Harris-Warrick and Cohen, 1985; Christenson et al., 1989; Kemnitz et al., 1995; Martin, 2002). 5-HT originates from three sources in lamprey: descending inputs from rhombencephalic neurons in the brainstem that run in the lateral tract; an intrinsic ventromedial spinal cord plexus; and fibers entering via the dorsal root ganglion (Cohen et al., 2005). Knowledge of the pharmacology of 5-HT receptors in lamprey lags that in mammals, where 5-HT_1A_ and 5-HT_2A,C_ receptors have been implicated in either improved functional recovery or pathogenesis after spinal lesions (e.g., Gimenez y Ribotta et al., 1998; Giroux et al., 1999; Hains et al., 2001; Hochman et al., 2001; Antri et al., 2003; Murray et al., 2010; Kong et al., 2011; see Gackière and Vinay (2014) for a recent review). Currently we know that the 5-HT-mediated reduction of the post-spike sAHP and fictive locomotor frequency in unlesioned mature adult animals seem to be mediated by a 5-HT_1A_ or 5-HT_2_ – like receptor, as the effects were mimicked by agonists of both of these receptors and blocked by the spiperone (a 5-HT_1A_ or 5-HT_2_ antagonist), but they were not blocked by specific 5-HT_2_ antagonists: 5-HT_3_ and 5-HT_4_ agonists and antagonists were without effect (Wikström et al., 1995). As in mammals (Giroux et al., 1999; Otoshi et al., 2009), in the larval lamprey 5-HT immunoreactivity was significantly reduced below the lesion in the lateral tract and ventromedial plexus 10 weeks after lesioning, the time point examined here (Cohen et al., 2005). At 10 weeks there was an increase in 5-HT immunoreactivity in the ventromedial plexus immediately rostral to the lesion site, possibly due to sprouting of spared fibers (Cohen et al., 2005). These changes in 5-HT levels and regeneration did not correlate with the degree of functional recovery (Cohen et al., 1999; Christenson et al., 1989). While the pharmacology of 5-HT effects in the lesioned spinal cord is unknown, 5-HT_1A_ receptor levels are increased 1–3 weeks after lesioning immediately above and 1–7 weeks immediately below the lesion site (Cornide-Petronio et al., 2014; note that the lesions in the Cohen et al., 2005 study were more caudal lesions to those used here, and the Cornide-Petronio et al., 2014 lesions more rostral). The relevance of this transient receptor up regulation, which also occurs in the cat (Giroux et al., 1999), is currently unknown, but it may relate to the need for 5-HT during the recovery period suggested by the PCPA experiments.

Assuming no volume transmission across the lesion, there is an obvious potential for differences in 5-HT levels on either side of a lesion site. In lamprey this could lead to faster activity below, where 5-HT levels are reduced, and slower 5-HT-modulated activity above the lesion site (Harris-Warrick and Cohen, 1985). The differences in activity either side of the lesion site would require an intersegmental co-ordinating signal that ensures the activity is properly integrated: failure to do this could result in poor locomotor recovery (Cohen et al., 1999; Christenson et al., 1989). In this context it could be speculated that the reduction of 5-HT effects in lesioned animals could help to reduce potential 5-HT-driven disparities in frequency by reducing the overall influence of 5-HT. The functional changes in basic cellular and synaptic properties may also contribute to this effect. Accepting the assumption that the swimming frequency reflects the degree of excitatory drive (Brodin et al., 1985), the greater inhibition below the lesion site would reduce the frequency but the increased excitation above would increase it: this could help to offset the differences in frequency caused by differences in 5-HT levels. This could be tested by manipulating excitability in unlesioned and lesioned animals either side of the lesion site. While this can easily be done for fictive locomotion, the differences in 5-HT effects on phase lag in the intact and fictive conditions (Harris-Warrick and Cohen, 1985; Kemnitz et al., 1995) raise the issue of how well fictive activity represents normal function (see also discussion of fictive activity in Ayers et al., 1983; Wang and Jung, 2002; Parker and Srivastava, 2013). Also, the discussion above only considers differences in 5-HT: a wide range of transmitters are released from descending neurons whose levels could differ either side of the lesion (Brodin et al., 1988). Differences in the effects of these individual transmitters will also have to be considered, as well as interactions between them.

A major issue is to place the differences in 5-HT effects in lesioned animals into a functional context. While this lack of understanding is an obvious weakness, similar uncertainty exists over the effects of 5-HT in unlesioned animals despite several studies that examine these effects. 5-HT has numerous cellular and synaptic effects in unlesioned animals: it reduces somatosensory and reticulospinal-evoked EPSP amplitudes (Buchanan and Grillner, 1991; El Manira et al., 1997), and has varied cell and synapse-specific effects on locomotor network neurons (see Parker, 2006 for details). How these effects influence the fictive or actual locomotor output in unlesioned animals is unknown. This understanding relies on knowing the segmental and intersegmental network organization: while this has been repeatedly claimed to be characterized there are still significant gaps in our understanding of even the basic network architecture (see Parker, 2006, 2010). The diverse cellular and synaptic effects of 5-HT would then need to be considered. It is claimed that the effects of 5-HT on fictive activity are explained by a reduction of the slow calcium-dependent (K_Ca_) sAHP following an action potential or via activation of NMDA channels in the crossing (CC) inhibitory interneurons, which delays switching of activity between the two sides and thus increases the cycle period (e.g., Matsushima and Grillner, 1992). However, this argument is flawed: relating effects solely to the CC interneurons begs the question as 5-HT affects the sAHP in other cells than the CC interneurons in ways that could increase the frequency; cells typically generate a small number of spikes which limits the possibility of sAHP summation; and blocking the sAHP with apamin has no effect on fictive locomotion over most of the frequency range (it only slows activity with lower NMDA concentrations, and this seems to be associated with disruption of the activity; see Buchanan, 2001). Understanding the role of the changes seen after lesioning in any system will require a consideration of how the varied functional effects act within well-defined locomotor networks.

## Conclusions

Pharmacological, electrical stimulation, and training effects modulate distinct functions in experimental and clinical situations (Courtine et al., 2009; Harkema et al., 2011; van den Brand et al., 2012). Knowing the changes in the lesioned spinal cord, how they relate to the degree of recovery, and how they respond to manipulations should allow these approaches to be targeted to maximize beneficial effects. Promoting regeneration remains the dominant approach to recovery from SPI, but the changes that occur in the lesioned spinal cord, either directed or non-directed (Beauparlant et al., 2013), could alter the response of networks to restored inputs (Bradbury and McMahon, 2006; Nahar et al., 2012), making a consideration of these changes necessary even if regeneration is assumed to be the dominant factor. If recovery reflects the integrated effects of functional changes above, below, and across the lesion site, as seems likely, perturbation of any component will only show necessity, not sufficiency in recovery, and thus all factors need to be considered. While we have identified various changes after lesioning in the lamprey model in this and previous studies, we currently do not know how these effects influence recovery. In addition to understanding the mechanisms underlying these effects, we now need to understand how these effects act in a defined, re-organized locomotor network and from this how they influence behavioral recovery.

## Conflict of interest statement

The authors declare that the research was conducted in the absence of any commercial or financial relationships that could be construed as a potential conflict of interest.

## Abbreviations

5-HT: 5-hydroxytryptamine
CV: coefficient of variation
RMP: resting membrane potential
PCPA: p-Chlorophenylalanine
GABA: gamma-aminobutyric acid
EMG: electromyogram
sAHP: slow afterhyperpolarisation
SiIN: small ipsilateral inhibitory interneuron
LIN: lateral interneuron
ScIN: small crossing inhibitory interneuron
CCIN: crossed caudal interneuron.
